# DIC-Transformer: interpretation of plant disease classification results using image caption generation technology

**DOI:** 10.3389/fpls.2023.1273029

**Published:** 2024-01-25

**Authors:** Qingtian Zeng, Jian Sun, Shansong Wang

**Affiliations:** College of Computer Science and Engineering, Shandong University of Science and Technology, Qingdao, China

**Keywords:** transformer, image caption, image classification, plant diseases, Faster R-CNN

## Abstract

Disease image classification systems play a crucial role in identifying disease categories in the field of agricultural diseases. However, current plant disease image classification methods can only predict the disease category and do not offer explanations for the characteristics of the predicted disease images. Due to the current situation, this paper employed image description generation technology to produce distinct descriptions for different plant disease categories. A two-stage model called DIC-Transformer, which encompasses three tasks (detection, interpretation, and classification), was proposed. In the first stage, Faster R-CNN was utilized to detect the diseased area and generate the feature vector of the diseased image, with the Swin Transformer as the backbone. In the second stage, the model utilized the Transformer to generate image captions. It then generated the image feature vector, which is weighted by text features, to improve the performance of image classification in the subsequent classification decoder. Additionally, a dataset containing text and visualizations for agricultural diseases (ADCG-18) was compiled. The dataset contains images of 18 diseases and descriptive information about their characteristics. Then, using the ADCG-18, the DIC-Transformer was compared to 11 existing classical caption generation methods and 10 image classification models. The evaluation indicators for captions include Bleu1–4, CiderD, and Rouge. The values of BLEU-1, CIDEr-D, and ROUGE were 0.756, 450.51, and 0.721. The results of DIC-Transformer were 0.01, 29.55, and 0.014 higher than those of the highest-performing comparison model, Fc. The classification evaluation metrics include accuracy, recall, and F1 score, with accuracy at 0.854, recall at 0.854, and F1 score at 0.853. The results of DIC-Transformer were 0.024, 0.078, and 0.075 higher than those of the highest-performing comparison model, MobileNetV2. The results indicate that the DIC-Transformer outperforms other comparison models in classification and caption generation.

## 1 Introduction

Rapid and accurate detection of plant diseases is crucial for increasing agricultural productivity. Traditionally, agriculture professionals rely on manual diagnosis to identify plant abnormalities caused by disease (Al-Hiary et al., 2011). However, this approach requires significant human and material resources and is not realistic (Ngugi et al., 2021). In response to these challenges, the use of image processing technology for automated diagnosis of plant diseases has garnered increased attention (Boulent et al., 2019).

In recent years, there has been remarkable progress in image classification due to the emergence of deep learning and neural networks. Among them, convolutional neural networks (CNNs) have shown good performance in image classification (such as Sun et al., 2022; Singh et al., 2023). However, a good CNN requires a large amount of training data (Keshari et al., 2018). Unfortunately, in the field of agricultural plant disease identification, the available labeled data are of poor quality and limited in quantity (Singh et al., 2020). Therefore, the first challenge in the task of classifying agricultural plant diseases through image classification is introduced: how to enhance the model’s classification performance with a relatively small number of images.

**Figure 1:**
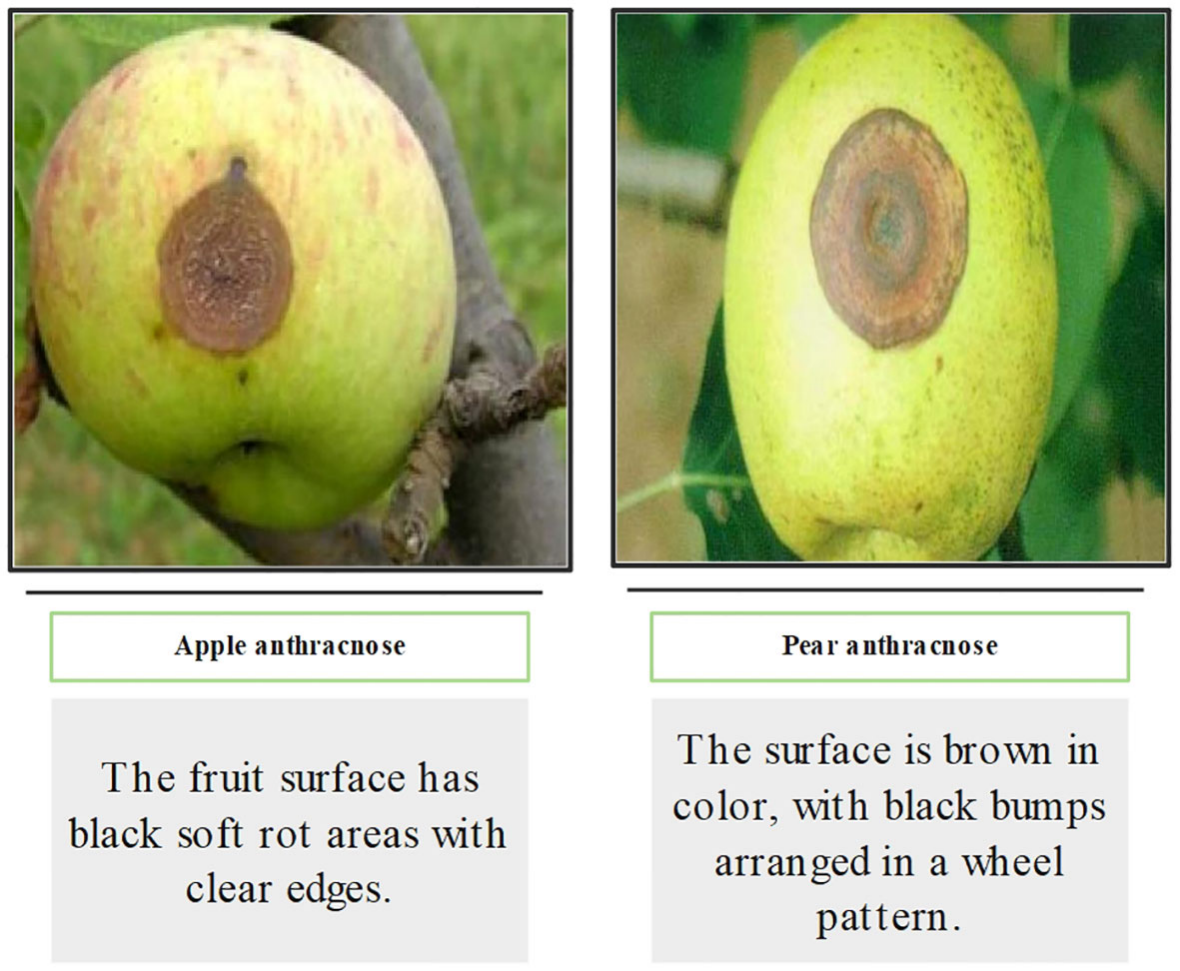
Examples of two diseases with similar pathogenic characteristics.

Among the existing agricultural diseases, some diseases have very similar pathogenic characteristics. For instance, “apple anthracnose” and “pear anthracnose” in **Figure 1** are challenging to differentiate based on visual features alone. As a result, the accuracy of CNN-based models in identifying similar disease classes is significantly reduced (Rzanny et al., 2022). However, this rarely occurs when agricultural professionals observe and confirm the disease. This presents a second challenge in identifying images of agricultural diseases: how to develop a model that can accurately identify specific plant diseases by replicating the process of manual disease diagnosis.

To address both of these challenges, this article introduces image caption generation techniques for the following reasons:

1. Image caption generation technology creates visual semantics based on features in disease images and then generates textual descriptions of affected areas using these visual semantics (Yang et al., 2022). This process closely resembles the behavior of agricultural experts manually identifying disease categories. This can address the second challenge presented.
2. The image classification task is single-task learning. When there are limited training data in the dataset, CNN-based image classification models may struggle to learn sufficient discriminant features, leading to slower performance in recognizing image classes (Luo et al., 2018). When there are limited image data samples, leveraging the benefits of multi-task learning can effectively mitigate the performance decline caused by the small sample size. Multi-task learning aims to extract useful information from other tasks and apply it to the current task, leading to an improved model performance model (Ruder, 2017). Image caption generation technology can extract rich semantic features from images (Xian et al., 2022). These visually and semantically rich features are shared during training, typically serving as discriminant features for recognizing objects. Therefore, by utilizing image caption generation technology, the model will continue to have a positive impact on image recognition even when there are relatively few image samples. Therefore, the combination of image captioning technology and image classification tasks addresses the first challenge. CNN-based image classification models may struggle to learn sufficient discriminant features, leading to slower performance in recognizing image classes (Luo et al., 2018). When there are limited image data samples, leveraging the benefits of multi-task learning can effectively mitigate the performance decline caused by the small sample size. Multi-task learning aims to extract useful information from other tasks and apply it to the current task, leading to an improved model performance model (Ruder, 2017). Image caption generation technology can extract rich semantic features from images (Xian et al., 2022). These visually and semantically rich features are shared during training, typically serving as discriminant features for recognizing objects. Therefore, by utilizing image caption generation technology, the model will continue to have a positive impact on image recognition even when there are relatively few image samples. Therefore, the combination of image captioning technology and image classification tasks addresses the first challenge.
3. By utilizing image caption generation technology, it is possible to combine images and text to create a multi-modal presentation. This tool is valuable for agricultural managers who need to analyze both visual and textual information simultaneously, allowing them to interpret agricultural plant disease classification results more comprehensively.

From the early methods to the recent advancements in deep learning, the accuracy of image caption generation has continuously improved. This has led to increased attention to the diversity of captions based on accuracy, which can generate more stylized image captions (Ghandi et al., 2023). In the field of agricultural diseases, the use of image caption generation technology is limited. While existing image recognition and classification technology for agricultural diseases is advancing, most models only provide the names of disease classes without clear explanations of the classification results (Kumar and Kumar, 2023). This lack of easy-to-understand explanations hinders farmers without specialized knowledge from correctly interpreting the recognition results, which does not align with real-world application needs. Given the current situation, this paper utilizes image caption generation technology in conjunction with image classification technology to produce descriptive information about disease characteristics based on the results of plant disease identification.

Based on image caption generation technology, a method called DIC-Transformer is proposed for agricultural plant disease image classification. This method can generate descriptive interpretations of disease areas in images.

The fourth section breaks down the method into four modules to accomplish three tasks: detection, interpretation, and classification. The four modules are the region detection module, sequence encoding module, caption generation module, and classification module. The disease region detection module completes the task of detecting the disease region. The second and third modules complete the interpretation task. The purpose of the interpretation task is to generate a textual description of the disease image features. The purpose of the classification task is to train a classifier that can identify categories of plant diseases. Finally, the name of the disease corresponding to the image is displayed. In the fifth section, the experimental part, we evaluate the DIC-Transformer on our self-constructed dataset (ADCG-18). The dataset contains 3,971 images, 9,040 instances of disease areas, and 3,971 textual descriptions. Experiments demonstrate that the DIC-Transformer method applied to the ADCG-18 dataset can address the proposed research topic. The article’s contributions are divided into the following three parts:

- A method for plant disease image classification is proposed. This method can output the name of the disease class and additionally generate an explanatory description of the characteristics of this disease class.
- An agricultural disease caption generation dataset is collected, named ADCG-18, which contains images of 18 diseases and textual descriptions of the corresponding images.
- Extensive experiments prove that the performance of the DIC-Transformer on the dataset ADCG-18 is the best.

This paper is divided into six chapters: Abstract, Introduction, Related Work, Dataset Construction, Methods, Experiments, and Conclusion. The Abstract introduces the limitations of existing methods, the structure of the DIC-Transformer, the dataset, and the experimental results. The Introduction outlines the article’s structure and the primary contributions. The Methods section outlines the principles and core concepts of DIC-Transformer. The Experimental section introduces the planned experiments, including the hardware configuration, software version, framework information, various parameters used, evaluation indicators, and the quantitative and qualitative analyses of the results. The Conclusion briefly outlines the issues addressed by our proposed model, its primary contributions, its limitations, and the future directions for improving the model.

## 2 Related work

### 2.1 Object detection

The continuous advancement of artificial intelligence, big data, and other technologies has ushered in new opportunities and challenges for image processing technology. The application of image processing technology in various fields is also more and more extensive and deep (Li et al., 2023). The rapid evolution of deep learning has also revolutionized the field of object detection. A large number of models of object detection use CNN models related to deep learning, such as R-CNN (Girshick et al., 2014), Fast R-CNN (Girshick, 2015), and Faster R-CNN (Ren et al., 2015). These methods improve detection performance by transforming object detection problems into classification and bounding box regression problems for candidate regions. In addition, these methods are essentially two-stage structures. After further development, single-stage detectors YOLO7 (You Only Look Once) (Wang et al., 2023) and SSD (Single Shot MultiBox Detector) (Liu et al., 2016) appeared. These methods reduce the complex candidate region generation process and improve the detection speed by predicting the class and location of the target directly on the image, but the detection accuracy in some scenarios is slightly inferior to that of the two-stage model. As object detection continues to evolve, researchers are constantly improving algorithms to improve performance. For example, attention mechanism, multi-scale feature fusion, and target shape information are introduced to enhance detection performance. **Table 1** summarizes the techniques related to objective detection, showing the name of the method, the time it was proposed, and the advantages and disadvantages of the method. Since the method proposed in this paper mainly requires the accuracy of the object detection module, the two-stage object detection model Faster R-CNN based on deep learning was selected as the disease area detection module of DIC-Transformer.

**Table 1:** Summary of work related to object detection.

Object Detection	Time	Advantages	Disadvantages
R-CNN	2014	SS is used to generate regions, and CNN is used to extract features	The training is multi-stage, the space consumption is large, and the detection is slow; duplicate calculations are performed at the time of feature extraction
Fast R-CNN	2015	The speed is higher than that of R-CNN; end-to-end training is achieved	An external SS module is required to generate the candidate region, which can only run on the CPU and is slow
SSD	2016	High accuracy, fast detection, small targets, and ease of use	Sensitive to target intensity; sensitive to dataset quality; performance degradation in complex scenarios
Faster R-CNN (Swin Transformer)	2022	High detection accuracy and great speed improvement, truly realize the end-to-end object detection framework	It takes a relatively long time
YOLO7	2022	Faster speeds, sufficient accuracy, and lower computing resource requirements	The accuracy is inflated; inaccurate detection of small targets; not versatile enough

### 2.2 Image caption generation

Image caption generation is a task that involves generating human-like descriptions or captions for given images. By generating textual descriptions related to the image, additional semantic information can be provided, enabling the computer to understand the image content more fully and deeply. Image captioning models generally come in three classes: template-based (TB), retrieval-based, and artificial neural network (ANN)-based (Bai and An, 2018).

The template-based (TB) model is a traditional image caption model, which requires the appropriate title structure to be defined in advance. Kulkarni et al. (2013) proposed a method called Babytalk, which combines computer vision and natural language processing techniques. First, they used computer vision algorithms to extract key visual features from images, such as objects, people, and scenes. They then used these features as input to generate simple descriptions related to the image through natural language processing models. However, image captioning technology based on TB mode can only detect image content visually, which generally causes problems such as complexity, creativity, and extracted sentence coverage. In addition, unlike the manually written image title information, if the main structure of the caption is a constrained template, it will make the generated caption a bit unnatural (Deorukhkar and Ket, 2022).

The retrieval-based image caption generation technology retrieves a sentence or set of sentences from a pre-defined text description dataset based on a given query image to generate the title that best matches the image features of the target image. The generated caption can be either a defined statement or a statement composed of multiple retrieved statements (Bai and An, 2018). Hodosh et al. (2013) treated caption generation as a ranking task. Captions and images are then mapped into the latent space based on canonical relevance. Also, the top-ranking caption for the target image is selected by calculating the cosine similarity between the caption and the image. In addition, two developments in caption retrieval were proposed by Ordonez et al. (2016) to score the relationship between captions and images. The two developments are as follows: one is the retrieval of the entire available image, and the other is the retrieval of captions based on the geometric distance of scenes and objects. In Jeong et al. (2023), a novel search-type radiology report generation module called X-REM is proposed to improve clinical accuracy. Compared to the benchmark retrieval method, X-REM increases the number of zero error reports and reduces the average error severity.

ANN-based models use an encoder–decoder architecture when generating image captions. Images are first encoded to generate a corresponding high-level representation and then decoded using language modeling algorithms. There are two types of ANN-based models: 1) recursion-based models and 2) transformer-based models (Parvin et al., 2023).

The recursive encoder–decoder structure is widely used in multiple tasks, such as machine translation, language generation, and code generation. Among them, long short-term memory network (LSTM) (Naga Srinivasu et al., 2023) and gated recurrent units (GRUs) are neural network units that are often used to construct recursive structures. For example, Ye et al. (2018) utilized attention mechanisms and linear transformations to improve image caption generation. Through steps such as calculating similarity, normalizing processing, and weighted summation, dynamically focus on image areas to generate more accurate and coherent captions. Yang et al. (2018) introduced a shared backbone network that is used to extract image features. Then, on top of the backbone network, multiple domain-specific task networks are built to process image caption generation tasks in different fields. Each task network has its own independent decoder for generating captions for the corresponding domain.

However, the recursion-based encoder–decoder architecture needs to be generated word by word in the process of generating the caption sequence, so the parallel computation is not possible, resulting in a slower speed (Khan et al., 2022). Due to the nature of the self-attention mechanism in the Transformer model, the representation of each position can be computed simultaneously with other locations without the need for sequential loop structures. This makes the Transformer model highly parallel computing power (Khan et al., 2022). At the same time, the transformer model consists of multiple stacked encoder and decoder layers, each with multiple self-attention sublayers and feedforward neural network sublayers. This multi-layered structure enables the gradual extraction of higher-level abstract features and more accurate predictions. These features make the Transformer-based caption generation model have advantages in handling long-distance dependency, parallel computing, and abstract feature extraction. They enable the model to better understand the image content and produce accurate, smooth captions. In the study of Wang et al. (2020), a geometry perception converter is constructed to obtain the geometric representation capabilities of encoders and decoders. Liu et al. (2021a) proposed a full-network structure CPtr based on Transformer for image caption generation tasks. By combining image feature representation with position encoding through an encoder, the decoder uses multiple Transformer layers to produce accurate captions. The attention mechanism is used to interact with the image with the text to improve the modeling ability. CPtr has shown excellent performance in image understanding and caption generation. In the study of Parvin et al. (2023), a transformer-based image description generative model is proposed that does not rely on recurrent or convolutional neural networks and is able to capture the interrelationships between objects. Experiments on COCO and Flickr datasets prove that the proposed method outperforms various state-of-the-art models in various evaluation indicators. In the study of Fang et al. (2022), ViTCAP, an image captioning model based on a pure visual transformer, is proposed, in which a grid representation is used without extracting regional features. In the study of Fei (2022), an attention-aligned converter for image captions is proposed, called A^2^, which is a perturbation-based, self-supervised way to guide attention learning without any annotation overhead. In the study of Liu et al. (2022), a new model based on the encoder–decoder framework is proposed. In the encoder, the features of different layers in the ResNet-50 are fused to extract multi-scale information. In the decoder, a multi-layer aggregation converter (MLAT) is proposed to utilize the extracted information to fully generate sentences. In the study of Jing and Jin-guang (2023), a hybrid structure image caption generation model based on a convolutional neural network and Transformer was proposed. It mainly fuses lightweight high-precision attention with convolutional networks to form attention residual blocks, which are used to extract visual features from input images. The features are then entered into the sequence model transformer. **Table 2** is a summary of the related technologies for image caption generation and lists the proposed time, advantages, and disadvantages of different methods. Then, the appropriate type of method is chosen according to the actual needs. Transformer architecture is first used in natural language processing and later widely used in the field of computer vision. Many transformer-based image captioning models were introduced in the study of Ondeng et al. (2023), including those pre-trained using visual language, which has produced several state-of-the-art models, etc. Recent models show the Transformer’s advantages in image caption generation. Therefore, the image caption generation model in this article used a transformer-based structure.

**Table 2:** Summary of work related to image caption generation.

Image caption	Method	Time	Advantages	Disadvantages
Template-based	Babytalk	2013	Descriptions can be accurately generated.	The content is single and relatively fixed, and the degree of human involvement is high.
Retrieval-based	X-REM	2023	Improved accuracy; simple to implement.	Performance depends on dataset size and retrieval algorithms; The generated descriptions are also relatively limited.
ANN-based	ALT MLADIC CPtr DPP ViTCAP A^2^ MLAT	2018 2018 2021 2022 2022 2022 2022	Automatically learn features in images; high flexibility and generalization ability; strong contextual comprehension.	High data demand; long training time; it is difficult to explain the specific decision-making process of the model for the generation of image captions.

## 3 Dataset construction

### 3.1 Image collection and preprocessing

To establish a plant disease image dataset, it is first necessary to determine which plants and diseases are selected. Then, high-quality images are collected, and finally, the images are preprocessed according to the requirements. The specific process is as follows:

1. Determination of plant species and diseases: Before collecting images, it is first necessary to determine the plant species and diseases. The difficulty of image collection is taken into account when determining plant species and diseases. If a plant disease can be searched for more images and related image characterization information through common search engines, then the disease is listed as a candidate. Finally, 18 diseases belonging to 10 plant species are selected as research objects to construct the dataset, for example, common plants such as apples, pears, and tomatoes.
2. Image collection: Images are collected in a variety of ways, such as search engine downloads, web crawling, and manual collection. The image collection is mainly based on web crawling and search engine downloads, supplemented by manual collection. In the end, more than 50,000 candidate images are collected. At the same time, the collection of images complies with relevant regulations such as copyright and privacy.
3. Image preprocessing: More than 50,000 images need to be processed to ensure that they are fit for use. Disease-affected areas in plant disease images need to have clear outlines, obvious symptoms, appropriate exposure, and other features. Low-quality, blurry, or unclear images need to be removed. The image filtering process is as follows:
  - Auto-filtering: For images that are not related to agricultural plant diseases, such as people, watermarks, and text, we use deep learning models to identify and remove them.
  - Manual filtering: After automatic filtering, because the content of the image is generally very complex, some images still do not meet the requirements, and relevant agricultural professionals need manual screening to ensure that the dataset images are basic and suitable for use.

The filtered image is more suitable for use in terms of content and resolution. In addition, most of the images are based on different backgrounds, closer to real agricultural scenes. Finally, a dataset containing 3,971 high-quality images for 18 diseases is constructed.

In summary, the ADCG-18 contains two modes, text and image, which have the characteristics of data diversity. Many different forms of data can enrich feature representation: images and text are two different forms of data that can provide complementary information to describe an object or scene. By using both image and text data, richer and more comprehensive feature representations can be obtained, which improves the performance and generalization ability of the model.

Data augmentation and transfer learning are also possible: fusing image and text data can augment the size and diversity of datasets to achieve data augmentation and improve the robustness and generalization of models. In addition, transfer learning between image and text data can help learning in one domain and improve the effectiveness of the model by helping another with what is learned in another. Therefore, it would be beneficial to consider both image and text data when building datasets. However, while data diversity is beneficial, there may be some potential biases, such as modal bias and data association bias. Modal bias is due to semantic differences between images and text, and models can produce bias when processing data with different modalities. For example, in image recognition tasks, the model may be more inclined to classify by image features and ignore the information described by text. Data correlation bias is the possibility of correlation bias when image and text data are combined through association rules or manual matching. Even if the association rule or matching process is deterministic, it is inevitable that there will be some errors or biases, resulting in the inaccurate correlation of images and text in the dataset.

These biases can affect the performance and generalization ability of the model, allowing the model to perform poorly against real-world samples. Therefore, when using datasets containing image and text information, it is necessary to pay attention to and minimize these potential biases and make corresponding preprocessing and adjustments to improve the robustness and accuracy of the model.

### 3.2 Data segmentation

**Figure 2:**
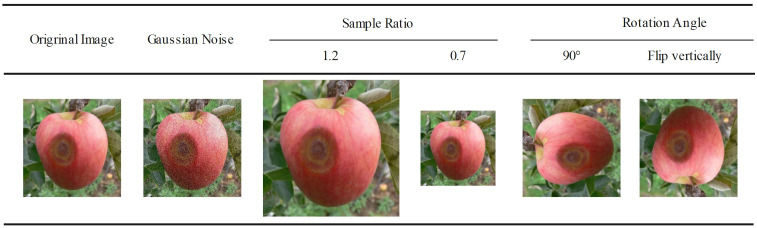
Examples of plant disease images of 18 types in the dataset.

The ADCG-18 contains 18 types of diseases due to the presence of data-enhanced similar images in the dataset, so it cannot be randomly divided according to the proportion when dividing the dataset. It is necessary to divide the images of each disease category according to the proportion and finally combine each divided disease data into the final dataset. Otherwise, the data images in the training set and the test set will be duplicated, resulting in higher experimental results than the real results. The data in this article are divided according to a 7:3 ratio. **Table 3** shows the results of the dataset division. The method of image enhancement is shown in **Figure 2**.

**Table 3:** Dataset segmentation results.

	Image_num	Instance_num
Test set	1,180	2,842
Train set	2,791	6,198
Total	3,971	9,040

Image num is the number of disease images. Instance_num is the number of disease instances.

### 3.3 Dataset details

The ADCG-18 contains two parts: images and textual descriptions. In addition, the images and textual descriptions in the dataset should correspond to each other. In order to achieve this, it is necessary to determine which descriptive keywords are unique to a certain disease class and ensure that the characteristic keywords in the textual description of each disease are clearly distinguished from the others.

Collect the description of diseases in the dataset from Internet resources such as relevant agricultural websites and then artificially extract sentences that meet the requirements for use. Finally, the textual description of each image consists of approximately six to 14 words. Note that each image corresponds to one textual description.

**Figure 3:**
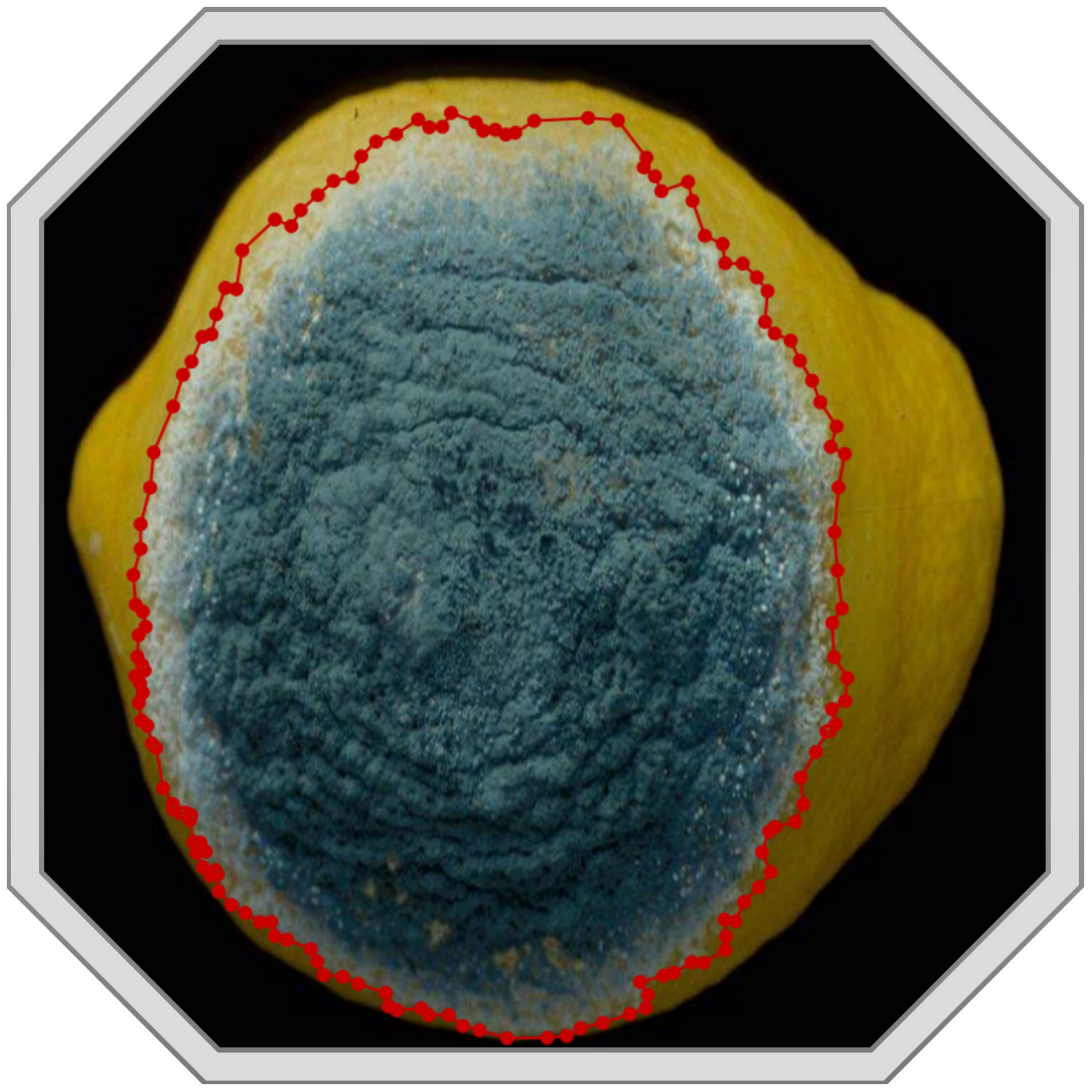
An example image processed by LabelMe.

Image labeling uses relevant tools to mark the disease area in the image, including the label of each disease category and the disease area’s true bounding box location. We use the image annotation tool LabelMe (Torralba et al., 2010) to manually mark the boundaries of the disease area in the image and save the boundary information in a JSON file. The version of LabelMe is 4.5.13. **Figure 3** shows the result of a hand-annotated image.

**Figure 4:**
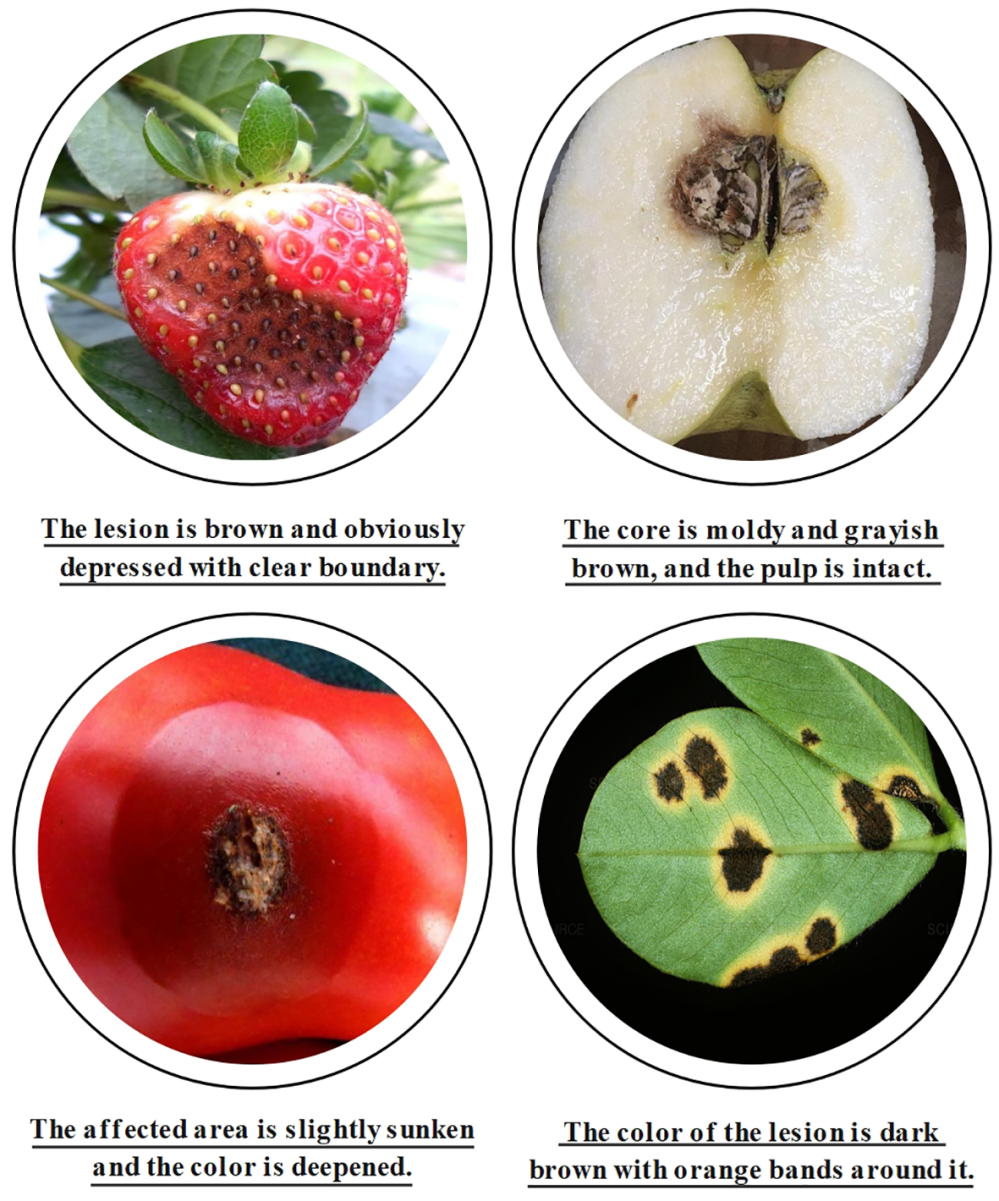
Dataset examples.

**Figure 5:**
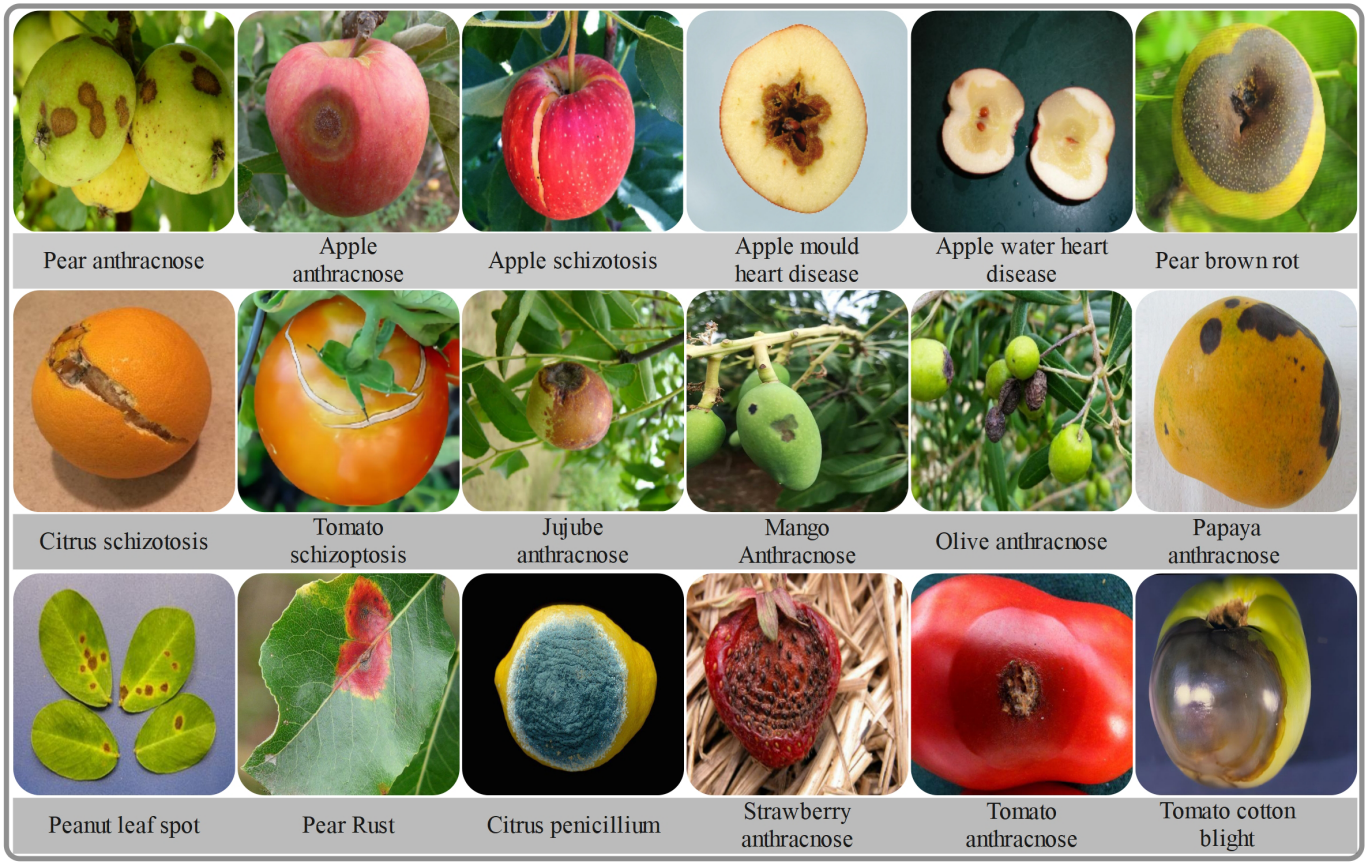
Examples of plant disease images of 18 types in the dataset.

Since at least one disease region exists per image, there are more disease instances (NOAD) than images (NOAI) for each class. Since the symptoms of each disease are different, the number of bounding boxes for each disease varies greatly. For example, in **Table 4**, Infection of Peanut_leaf_spot and Pear_Rust are characterized by the presence of more small-area spots on the foliage, and the number of instances is 1,478 and 951, respectively, which is much higher than the average number of instances. **Figure 4** shows some image samples and corresponding textual descriptions. **Figure 5** shows the characteristic images of 18 diseases.

**Table 4:** Statistical analysis of datasets.

SN	Category name	NOAI	NOAD	NOTRI	NOTRD	NOTI	NOTD
1	Anthrax	165	344	116	219	49	125
2	Apple_bitter_rot	252	408	176	270	76	138
3	apple_cracking	156	248	110	175	46	73
4	Apple_mould_heart_disease	195	277	135	177	60	100
5	Apple_water_heart_disease	230	898	162	684	68	214
6	Brown_rot	261	289	173	184	88	105
7	Citrus_schizoderma	381	557	291	324	90	233
8	cracking	371	673	263	388	108	285
9	Jujube_Anthrax	219	649	150	337	69	312
10	Mango_Anthracnose	217	343	152	258	65	85
11	Olive_anthracnose	115	234	77	137	38	97
12	Papaya_anthracnose	212	673	149	499	63	174
13	Peanut_leaf_spot	235	1,478	164	1,140	71	338
14	Pear_Rust	268	951	190	680	78	271
15	Penicillium	165	187	115	136	50	51
16	strawberry_anthracnose	174	204	121	139	53	65
17	Tomato_anthracnose	188	348	130	251	58	97
18	Tomato_cotton blight	167	279	117	200	50	79

SN, the category index; NOAI, the total number of images; NOAD, the total number of disease areas; NOTRI, the number of images in the training set; NOTRD, the number of disease areas in the training set; NOTI, the number of images in the test set; NOTD, the number of disease areas in the test set.

In addition, as depicted in **Figure 3**, we primarily describe the quantity, color, size, sharpness of boundary lines, brightness, darkness, and the degree of variation in characteristics when constructing the dataset. These characteristics encompass all the pathogenesis features of the disease and can fully describe the occurrence of the disease. The text is stored in a JSON file as a dictionary. Each entry begins with a disease name followed by a description of its features. It is important to note that image name labels have been included in the text dataset to link each statement to an image, which aids in the model’s training process.

## 4 Methods

### 4.1 Overview

**Figure 6:**
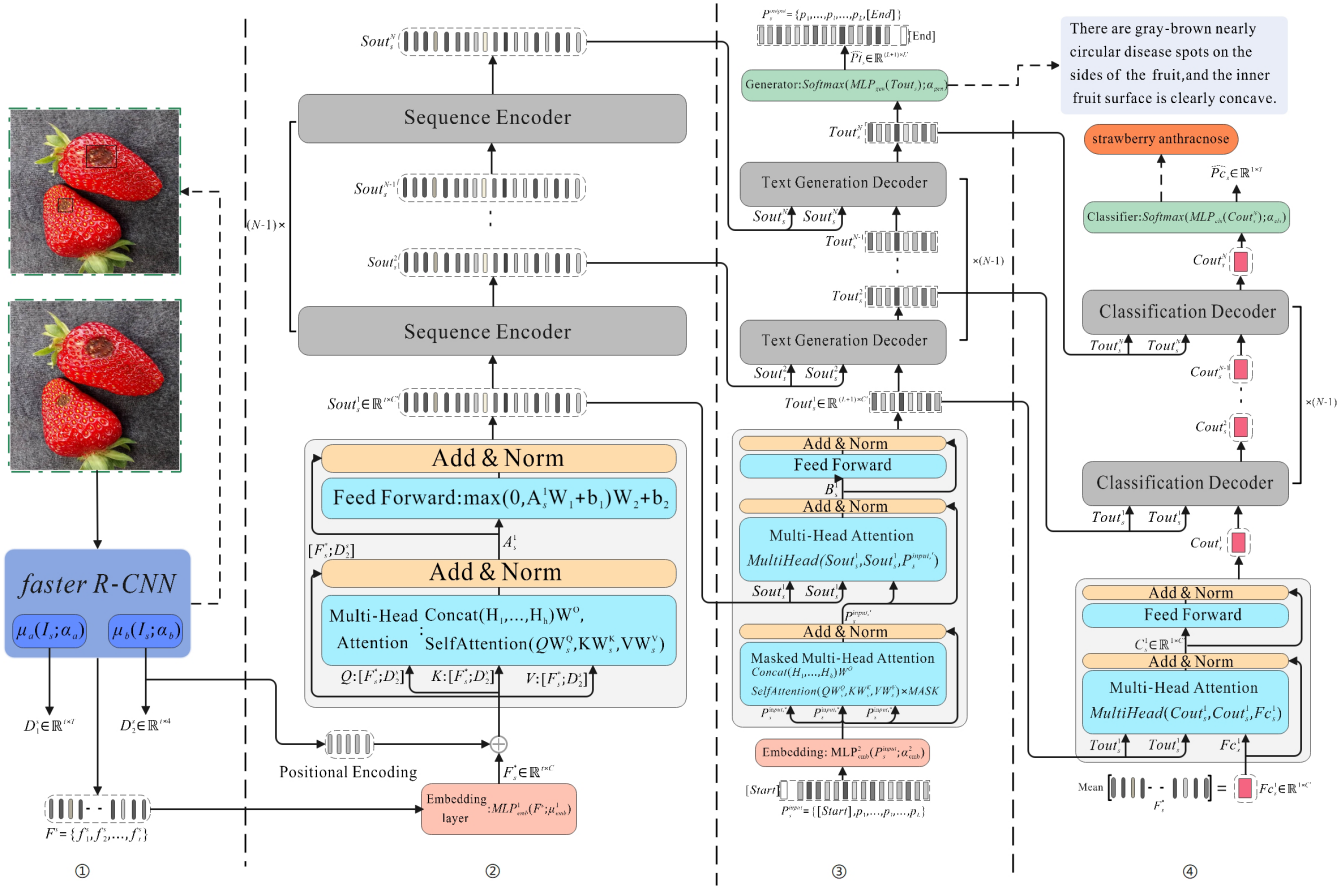
Model overview diagram. The numbers at the bottom of the figure represent four modules: ① represents the region detection module, ② represents the sequence encoding module, ③ represents the caption generation module, and ④ represents the classification module.

**Figure 6** shows the overall structure diagram of the model DIC-Transformer proposed in this paper, with the DIC-Transformer divided into four modules. ① is a disease region detection module, which is used to obtain relevant information about the disease area in the image. Relevant information is divided into two categories: location vectors and visual feature vectors of disease areas. ② and ③ are sequence encoding module and caption generation module, respectively, which are used for image caption generation tasks. ④ is a classification module designed to classify images. We divide these four modules into two stages, where the first stage model includes module ① and the second stage model includes modules ②, ③, and ④.

The first stage model, the region detection module, can acquire the label of the disease image and the vector representation of the disease area feature in the image. These feature vectors are integrated into the sequence as inputs to the sequence coding module. The average value of the integrated visual feature vector sequence is used to represent the entire image. The output of the sequence encoding module, in turn, serves as keywords and values for attention blocks in each text generation module. The input to the text generation module is a sequence of word vectors, and the first position of the sequence is the symbol [Start], which marks the beginning of generating the word vector sequence. The output of the text generation module is a sequence of word vectors similar to the input. The text generation module is like RNN one word input, the Q is calculated by the word that has appeared, the K and V are calculated by the sequence coding module, and the result is obtained after all the text generation modules and then FC+Softmax. After that, the result is used as input to the text generation module, and the whole process is repeated until the symbol END is output. The output of the END symbol at this point indicates that a text sequence has been generated. The output of each layer of the text generation module is a visual vector weighted by text features. Now, it is used as the keyword and value of the classification module, and the average value of the visual feature vector sequence is used as the query of the classification module. Finally, the output of the disease classification module is the probability for each category. In order to clearly describe the implementation details of each module, a symbology is established.

### 4.2 Region detection module

The region detection module is used to obtain the feature vectors and bounding box coordinates for each disease area in the image. Let $I\;=\;\left\{I_{1},...\;,I_{s},...\;,I_{S}\right\}$ denote the set of images, where *S* denotes the total number of images. Let $D^{s}\;=\left\{d_{1}^{s},d_{2}^{s}...\;,\;d_{t}^{s}\right\}$ denote disease areas that appear in each image *I_s_*. Each disease area contains two types of information: a) $d_{t,1}^{s}\in {\mathbb{R}}^{1\times T}$ represents the label of the diseased area, where *T* represents the total number of categories for disease areas, and b) $d_{t,2}^{s}\in {\mathbb{R}}^{1\times 4}$ represents the bounding box coordinates of each diseased area. Specifically, the region detection module learns two mapping functions: *µ_a_* and *µ_b_*. *µ_a_* is used to obtain the category labels of each disease area, while *µ_b_* is used to obtain the bounding box coordinates for each disease area. The details of the two functions are shown in Equations 1, 2.


(1) $${\hat{d}}_{*,2}^{s}=\mu_{b}\left(I_{s};\alpha_{b}\right),d_{*,2}^{s}\in {\mathbb{R}}^{t\times 4}$$

(2) $${\hat{d}}_{*,1}^{s}=\mu_{a}\left(I_{s};\alpha_{a}\right),d_{*,1}^{s}\in {\mathbb{R}}^{t\times T}$$

where *α_a_* represents the weight of the function *µ_a_* and *α_b_* represents the weight of the function *µ_b_*. Area ① in **Figure 6** represents the region detection module, where $F^{s}\;=\;\{f_{1}^{s},f_{2}^{s},\ldots ,f_{t}^{s}$ represents the features of diseased areas in the image. These features are obtained from the feature map generated by the last convolutional layer of the backbone network in the object detection process. Specifically, $f_{t}^{s}\in {\mathbb{R}}^{1\times C}$ represents the feature vector of a specific disease area in the image. The default value for *C* is 1,024.

### 4.3 Sequence encoding module

The structure of the sequence encoding module is shown in area ② in **Figure 6**. The intermediate variable of the function *µ_a_* in the region detection module is used as input to the sequence encoding module, where the intermediate variable is essentially the visual vector generated by the integration of all disease regions in the image. This collection of visual vectors is referred to as the visual vector sequence, denoted as $F^{s}=\left\{f_{1}^{s},f_{2}^{s},\;.\;.\;.\;,f_{t}^{s}\right\}$ , where $F^{s}\in {\mathbb{R}}^{t\times C}$ . To encode this sequence of visual vectors, an input embedding layer is utilized, which consists of two fully connected layers with an output dimension of C' , having a default value of 1,024. This process is represented by Equation 3.


(3) $$F_{s}^{*}=MLP_{emb}^{1}\left(F^{s};\alpha_{emb}^{1}\right),F_{s}^{*}\in {\mathbb{R}}^{t\times C^{\prime }}$$

where $\alpha_{emb}^{1}$ is the weight of $MLP_{emb}^{1}$ .

The self-attention mechanism can be regarded as an improvement mechanism for the attention mechanism in a certain application scenario. It becomes less dependent on external information and has a superior performance in capturing internal correlations in data or features. The calculation process of the self-attention mechanism is as follows: first, the input data is converted into an embedding vector. According to the embedding vector, the three vectors of $Query\left(Q=\;\{q_{1},\ldots ,q_{s}\}\right)$ , $Key\left(K=\;\{k_{1},\ldots ,k_{t}\}\right)$ , and $Value\left(V=\;\{v_{1},\ldots ,v_{t}\}\right)$ are obtained. Calculate a score for each vector. To ensure gradient stability, use score normalization by dividing by., where $\sqrt{Y}$ is a scaling factor to prevent the input values for the softmax function from becoming too large. Apply a softmax activation function to score. The specific process is represented by Equation 4.


(4) $$\text{Self Atention}\left(Q,K,V\right)=Softmax(\frac{QK^{T}}{\sqrt{Y}})V$$

where $Q\in {\mathbb{R}}^{s\times a},K\in {\mathbb{R}}^{t\times a},V\in {\mathbb{R}}^{t\times a}$ .

The attention mechanism is position-insensitive, and even swapping the position of two elements in the sequence has no effect on the encoded result. Therefore, a positional vector notation is proposed in the Transformer to add a fixed positional vector to each position of the sequence. However, the visual vector sequences in this paper only have a spatial position relationship and no context relationship. Therefore, for the use of positional encoding, we use the four coordinates of the visual bounding box as positional encoding, discarding the original sine cosine function. In addition, the multi-head self-attention mechanism is used to study the relationships within the visual vector sequence. The self-attention mechanism is repeated *h* times in the long self-attention mechanism. The specific process is represented by Equation 5.


(5) $$\begin{array}{l} MultiHead\left(Q,K,V\right)=\mathrm{Concat}\left(H_{1},\ldots ,H_{h}\right)W^{O},\\ H_{s}=\text{Self Atention}\left(QW_{s}^{Q},KW_{s}^{K},VW_{s}^{V}\right) \end{array}$$

where $W^{O}\in {\mathbb{R}}^{a\times a}$ and $W_{s}^{Q},W_{s}^{K},W_{s}^{V}\in {\mathbb{R}}^{a\times \frac{a}{h}}$ .

The sequence coding module consists of two sublayers: the multi-head self-attention layer and the feedforward neural network layer. Each sublayer is followed by the AddNorm function. The AddNorm function is a common regularization operation commonly used in neural networks. It combines residual connectivity and layer normalization to enhance the representation and training effect of the network. The calculation process of the sequence encoding module is shown in Equations 6, 7.


(6) $$A_{s}^{1}=AddNorm\left(MultiHead\left(\left[F_{s}^{*};D_{2}^{s}\right],\left[F_{s}^{*};D_{2}^{s}\right],\left[F_{s}^{*};D_{2}^{s}\right]\right)\right)$$

(7) $$Sout_{s}^{1}=AdNorm\left(FFN\left(A_{s}^{1}\right)\right)=AddNorm\left(max\left(0,A_{s}^{1}W_{1}+b_{1}\right)W_{2}+b_{2}\right)$$

where $A_{s}^{1}\in {\mathbb{R}}^{t\times C^{\prime }},W_{1},W_{2}\in {\mathbb{R}}^{C^{\prime }\times C^{\prime }},b_{1},b_{2}\in {\mathbb{R}}^{C^{\prime }},Sout_{s}^{1}\in {\mathbb{R}}^{t\times C^{\prime }}$ . The output of each layer of the encoder in the sequence encoding block is $Sout_{s}^{N}\in {\mathbb{R}}^{t\times C^{\prime }}$ , N represents the number of encoders in the sequence encoding module.

### 4.4 Caption generation module

Area ③ in **Figure 6** shows the structure of the caption generation module. This module predicts the next word based on the input word and ensures that the resulting sentence visually corresponds to the disease area in the image. Prediction begins upon recognizing the marker [*Start*]} and concludes when the marker [*End*]} is generated. Both [*Start*]} and [*End*]} are zero vectors. The input to the caption generation module is denoted as $P_{s}^{input}=\left\{\left[Start\right],\;p_{1},\;.\;.\;.\;,\;p_{l},\;.\;.\;.\;,\;p_{L},\right\}$ , where $P_{s}^{input}\in {\mathbb{R}}^{(L+1)\times L'}$ represents the description statement corresponding to the visual part *D_s_*. The output of the caption generation module is represented as $P_{s}^{output}=\left\{p_{1},\;.\;.\;.\;,\;p_{l},\;.\;.\;.\;,\;p_{L},\;\left[End\right]\right\}$ , where $P_{s}^{output}\in {\mathbb{R}}^{(L+1)\times L'}$ . Here, *L* is the length of the input statement and L' is the number of words in the database.

The input statement of the module needs to be encoded by the word embedding layer, which consists of two fully connected layers with an output dimension of C' . The embedding layer process is represented by Equation 8.


(8) $$P_{s}^{input,*}=MLP_{emb}^{2}\left(P_{s}^{input};\alpha_{emb}^{2}\right),P_{s}^{input}\in {\mathbb{R}}^{(L+1)\times C^{\prime }}$$

where $\alpha_{emb}^{2}$ is the weight of $MLP_{emb}^{2}$ .

The decoder model consists of N identical decoder blocks stacked, each consisting of three different sublayers. There are residual connections and layer normalization between each sublayer. Moreover, the masked multi-head self-attention sub-layer uses a mask to prevent information from future output words from being used when training a given output word. The form of MASK is shown in Equation 9.


(9) $$MASK=\left[\begin{array}{cccc} 1 & 0 & \cdots & 0\\ 1 & 1 & \cdots & 0\\ \vdots & \vdots & \ddots & \vdots \\ 1 & 1 & 1 & 1 \end{array}\right],MASK\in {\mathbb{R}}^{\left(L+1\right)\times \left(L+1\right)}$$

Thus, the process of self-attention with masking can be expressed as Equation 10.


(10) $$\begin{array}{l} MaskMultiHead\left(Q,K,V\right)=Concat\left(H_{1},\ldots ,H_{h}\right)W^{O}\\ H_{s}=Self\;Attention\left(QW_{s}^{Q},KW_{s}^{K},VW_{s}^{V}\right)\times MASK \end{array}$$

where $W^{O}\in {\mathbb{R}}^{a\times a}$ and $W_{s}^{Q},W_{s}^{K},W_{s}^{V}\in {\mathbb{R}}^{a\times \frac{a}{h}}$ .

The first layer decoder in the caption generation module is taken as an example, and formulas are used to describe the working process of the decoder. The input encoded by the embedding layer is first copied three times and then input into a masked self-attention block to obtain an output, which can be represented by Equation 11.


(11) $$P_{s}^{input{,}^{\prime }}=AddNorm\left(maskMultiHead\left(P_{s}^{input,*},P_{s}^{input,*},P_{s}^{input,*}\right)\right)$$

where $P_{s}^{input{,}^{\prime }}\in {\mathbb{R}}^{(l+1)\times C'}$ . The output $Sout_{s}^{1}$ of the first-layer encoder in the sequence encoding module is *K*, *V*, and $P_{s}^{input{,}^{\prime }}$ is *Q*. Then, *Q*, *K*, and *V* are used to calculate multi-head self-attention, the purpose of which is to explore the implicit relationship between visual features and semantic features, essentially using visual vectors weighted by text features to generate descriptive statements. The specific process can be represented by Equations 12, 13.


(12) $$B_{s}^{1}=AddNorm\left(MultiHead\left(Sout_{s}^{1},Sout_{s}^{1},P_{s}^{input{,}^{\prime }}\right)\right)$$

(13) $$Tout_{s}^{1}=AddNorm\left(FFN\left(B_{s}^{1}\right)\right)$$

where $B_{s}^{1}\in {\mathbb{R}}^{(L+1)\times C^{'}}$ . $Tout{\;}_{1}^{s}\in {\mathbb{R}}^{(L+1)\times C^{\prime }}$ represents the output of the first layer decoder of the caption generation module.

The caption generation module contains a total of N decoders, and the output of the last layer decoder is $Tout{\;}_{s}^{N}$ . Then, two fully connected layers are used to convert the output $Tout_{s}^{N}$ into the distribution probability of each word in the database. This probability can be expressed using Equation 14.


(14) $${\hat{Pt}}_{s}=Softmax\left(MLP_{gen}\left(Tout_{s}^{N}\right);\alpha_{gen}\right)$$

where ${\hat{Pt}}_{s}\;\in {\mathbb{R}}^{(L+1)\times L^{\prime }}$ is the weight of *MLP_gen*.

### 4.5 Classification module

The structure of the classification module is shown in area ④ in **Figure 6**. The purpose of the classification module is to predict disease image categories based on the output of the caption generation module. The output of the caption generation module serves as *K* and *V* for attention blocks in the classification decoder. Its output is essentially a visual vector weighted by text features. In addition, the classification module and the caption generation module have the same number of decoders. Let $Fc_{s}^{1}\;=\;Mean(F_{s}^{*}),\;Fc_{s}^{1}\;\in {\mathbb{R}}^{1\times C^{'}}$ denote a complete image of an agricultural disease, which is taken as the *Q* in the attention block. Taking the first attention block in the classification module as an example, the operation process is represented by Equations 15, 16.


(15) $$C_{s}^{1}=AddNorm\left(MultiHead\left(Tout_{s}^{1},Tout_{s}^{1},Fc_{s}^{1}\right)\right)$$

(16) $$Cout_{s}^{1}=AddNorm\left(F\;F\;N\left(C_{s}^{1}\right)\right)$$

where $C_{s}^{1}\in {\mathbb{R}}^{1\times C^{\prime }}$ . $Cout_{s}^{1}\in {\mathbb{R}}^{1\times C^{\prime }}$ is the output of the first decoder in the classification module. Let $Cout_{s}^{N}\in {\mathbb{R}}^{1\times C^{\prime }}$ denote the output of the last decoder. The output $Cout_{s}^{N}$ of the last decoder is then converted into a probability distribution of the agricultural disease image category through two fully connected layers. The probability equation is shown in Equation 17.


(17) $$\hat{P_{c_{s}}}=\;Softmax\left(MLP_{cls}\left(Cout_{s}\right);\;\alpha_{cls}\right)$$

where ${\hat{Pc}}_{s}\in {\mathbb{R}}^{1\times T}$ , *T* is the number of disease categories, and *α_cls_* is the weight of *MLP_cls_*.

## 5 Experimental

### 5.1 Experimental content

The DIC-Transformer mainly includes three modules: disease region detection module, image caption generation module, and classification module. Then, the results of Faster R-CNN experiments in 16 different backbones are analyzed, and an optimal object detection model is selected to process the input images. Next, the second task, the image caption generation task, needs to be tested and analyzed, and its main job is to compare DIC-Transformer with some existing caption generation models. Finally, we need to evaluate the performance of the classifier. Since DIC-Transformer is a two-stage method, the experiment mainly consists of two tasks:

1. Object detection backbone comparison experiment based on Faster R-CNN.
2. Quantitative and qualitative analyses of the DIC-Transformer. This task is divided into the following four experiments:
  - Comparative experiment of image caption generation model.
  - Comparative experiments of classification models.
  - Ablation experiments of DIC-Transformer.
  - Qualitative analysis of DIC-Transformer and classic image caption generation models.

### 5.2 Experimental details

Object detection backbone comparison experiments based on Faster R-CNN are implemented in two open-source frameworks, Detectron2 (Wu et al., 2019) and MMDetection (Chen et al., 2019). Detectron2 is a robust object detection platform developed by FAIR (Facebook AI Research) in 2019. Several state-of-the-art detection and segmentation algorithms are already integrated, eliminating the need to develop these networks from the ground up. There are two types of object detectors: one-stage and two-stage detectors. Detectron2 is a two-stage system, and the detection task is carried out in two steps. The first step is to extract the region of interest (RoI). The second step involves target classification and positioning. The nature of these two-stage detectors makes them slower than one-stage detectors such as YOLO and SSD, but they can produce more accurate results. Under Detectron2, we utilize the Faster R-CNN + FPN algorithm and employ a pre-trained model. In Detectron2, there are no epochs, only iterations. However, the maximum number of iterations can be artificially set based on the size of the dataset.

MMDetection is an open-source project initiated by SenseTime and the Chinese University of Hong Kong for object detection tasks. It implements a wide range of object detection algorithms based on PyTorch and encapsulates the processes of dataset construction, model building, training strategies, and other tasks into modules. When building a new algorithm with MMDetection, the process typically involves the following steps: registering the dataset, registering a model, building a configuration file, and conducting training and validation.

The second stage of the DIC-Transformer is trained and tested on an NVIDIA P100-16G with CUDA 11.3 using Python 3.8 and PyTorch 1.10 on Ubuntu 18.04. Additionally, the version of Detectron2 is v0.6, and the version of MMDetection is 2.25.1.

The parameters used in the experimental process are detailed in **Table 5**. Both frameworks employ the same parameter settings, where unexposed parameters utilize the default values within the framework.

**Table 5:** Faster R-CNN benchmark experiment parameter setting.

Parameter	Pre-trained model	Optimizer	Warm-up strategy	Warm-up iters	Warm-up ratio
Value	ImageNet	SGD	Liner	1,000	0.001
Parameter	BatchSize	Learning rate	Momentum	Weight decay	Iteration rounds
Value	16	0.02	0.9	0.0001	10,000

The region detection module is used to extract the feature and location of the disease area as the input of the caption generation module. The parameters for all comparison and ablation experiments are shown in **Table 6**.

**Table 6:** Comparative experiment parameter settings for DIC-Transformer.

Parameter	Noamopt Warmup	Optim Alpha	Optim Beta	Optim Epsilon	Noamopt	Optimizer
Value	Adam	0.9	0.999	1e−4	True	2,000
Parameter	Noamopt Factor	Grad Clip	BatchSize	Learning Rate	T-Depth	Epoch
Value	1	0.1	16	2e−4	6	30

The second stage of the DIC-Transformer is trained and tested on NVIDIA P100-16G with CUDA 11.3 using Python 3.8 and PyTorch 1.10 on Ubuntu 18.04. Additionally, the Detectron2 version used is v0.6, and the MMDetection version is 2.25.1.

### 5.3 Evaluation metrics

We mainly evaluate three tasks: object detection task, image caption generation task, and image classification task. Object detection task uses *mAP*/*mAP*_50_/*mAP*_75_ as quantitative indicators. The image caption generation task uses BLEU, Cider-D, and Rouge as quantitative indicators. Image classification task uses Acc, Recall, and F1 as quantitative indicators. Some basic concepts of evaluation indicators are as follows.

#### 5.3.1 IoU

This represents an intersection over the previous union, essentially the overlap between the predicted range and the true range divided by the sum of the predicted range and the true range. Equation 18 is the calculation process of the IoU.


(18) $$IoU=\frac{P\cap^{\;}G}{P\cup^{\;}G}$$

where *P* is the predicted bounding box and *G* is the ground-truth bounding box.

#### 5.3.2 Confusion matrix

This is a summary of the prediction results, where TP represents the number of predictions that will be positive to positive classes. FN differs from TP in that it is the number of positive classes predicted as negative classes. FP is the exact opposite of FN, and it is the number of negative classes predicted as positive classes. The final TN is the exact opposite of TP, which represents the number of predicted negative classes as negative classes. In the taxonomic issue, for a disease category like strawberry anthracnose, the sample labeled “strawberry anthracnose” is considered a positive sample, while all other samples are considered negative. Therefore, in the classification problem, TP represents the image predicted by the model as strawberry anthracnose, and the true label is also strawberry anthracnose, i.e., the number of samples correctly predicted as positive. FN represents the image that the model predicts to be non-strawberry anthracnose, while the true label is strawberry anthracnose, i.e., the number of samples that are falsely predicted as positive. TN represents the image that the model predicts to be non-strawberry anthracnose, while the true label is also not strawberry anthracnose, i.e., the number of samples correctly predicted to be negative. FP represents the image that the model predicts to be strawberry anthracnose, but the true label is not strawberry anthracnose, i.e., the number of samples that are incorrectly predicted as positive.

#### 5.3.3 Precision

It indicates the proportion of samples that are actually positive in a sample that is predicted to be positive. The calculation process is shown in Equation 19.


(19) $$precision=\frac{TP}{TP+FP}$$

#### 5.3.4 mAP

It measures the ability of the trained model to detect all classes. *mAP*/*mAP*_50_/*mAP*_75_ is *mAP^IoU^* ^=0.5:0.05:0.95^/*mAP^IoU^* ^=0.5^/*mAP^IoU^* ^=0.75^. *IoU* = 0.5:0.05:0.95 means that the intersection over union ratio is calculated for values ranging from 0.5 to 0.95, with an interval of 0.05. Equation 20 represents the calculation process of *mAP*.


(20) $$mAP=\frac{\sum_{i=1}^{K}AP_{i}}{K}$$

where *AP* is defined as the area between the interpolated precision-recall curve and the X-axis, *K* represents the number of categories, and *AP_i_* represents the *AP* value for a category.

#### 5.3.5 BLEU

What the BLEU algorithm is actually doing: judging how similar two sentences are. Equation 21 represents the implementation of BLEU.


(21) $$bleu_{n}=\frac{\sum_{c\in candidates}\;\sum_{n-gram\in e}\;Count_{clip}\left(n-gram\right)}{\sum_{c^{\prime }\in candidates}\;\sum_{n-gram^{\prime }\in e^{\prime }}\;Count\left(n-gram^{\prime }\right)}$$

where the purpose of the first summation symbol in the formula is to calculate all the candidates because there may be several sentences in the calculation. Then, the purpose of the second summation symbol is to count all n-gram in the candidate. The number of n-gram in the reference statement is denoted by $Count_{clip}\left(n-gram\right)$ . Thus, the numerator is the representation of how many n-gram appears in a given candidate reference statement. The number of n-gram′ in the candidate is represented by $Count\left(n-gram^{\prime }\right)$ . Therefore, the denominator is the number of n-gram among all candidates.

#### 5.3.6 Cider-D

The purpose of A is to prevent gaming problems with evaluation indicators. The problem with gaming is to prevent optimizing the algorithm for a certain evaluation indicator so that when the human gives a low score, the evaluation index gives a high score. Equations 22, 23 describe the calculation process for this evaluation metric.


(22) $$Clder-D_{n}\left(x_{i},y_{i}\right)=\frac{100}{m}\sum_{j}\;e\frac{-{\left(l\left(x_{i}\right)-l\left(y_{i,j}\right)\right)}^{2}}{2\delta^{2}}*\frac{\min\;\left({ɡ}^{n}\left(x_{i}\right),{ɡ}^{n}\left(y_{i,j}\right)\right)\cdot {ɡ}^{n}\left(y_{i,j}\right)}{||{ɡ}^{n}\leq ft(x_{i})||||{ɡ}^{n}\left(y_{i,j})|\right|}$$

(23) $$\text{Cider D }\left(x_{i},y_{i}\right)=\sum_{N}^{n=1}\;w_{n}CiderD_{n}\left(x_{i},y_{i}\right)$$

where *l*(*x_i_*) is the length of the text generated by the model and *l*(*y_ij_*) is the length of the real text. Multiplying by 100 makes the value of Cider-D similar in size to the value of other evaluation indicators. *g^n^* consists of *g*_1_, *g*_2…_ *g_n_*. *g_k_* is used to calculate the TF-IDF value for each N-gram. We set *δ* = 6 and N = 4.

#### 5.3.7 Rouge

Rouge metrics are very similar to BLEU metrics. The main difference is that ROUGE is based on recall, while BLEU focuses more on precision. The calculation process is shown in Equation 24.


(24) $$Rouge_{lcs}=\frac{\left(1+\beta^{2}\right)R_{lcs}P_{lcs}}{R_{lcs}+\beta^{2}P_{lcs}}$$

where *β* = 1.2. The calculation process for *R_lcs_* and *P_lcs_* is shown in Equations 25, 26, respectively.


(25) $$R_{lcs}=\frac{LCS\left(X,Y\right)}{m}$$

(26) $$P_{lcs}=\frac{LCS\;\left(X,Y\right)}{n}$$

where *X* represents the text generated by the model, and the length is *m*. *Y* represents the real text of the image, and the length is *n*. *LCS* is the longest common subsequence.

#### 5.3.8 Acc

The full name of ACC is Accuracy, which stands for accuracy. The accuracy can be expressed by Equation 27.


(27) $$Accuracy=\frac{TP+TN}{TP+TN+FP+FN}$$

#### 5.3.9 Recall

It indicates the proportion of correctly predicted true values among all positive cases, which can be understood as how many correct targets are recalled. The calculation process is shown in Equation 28.


(28) $$Recall=\frac{TP}{TP+FN}$$

#### 5.3.10 F1

The core idea of F1 is to improve Precision and Recall as much as possible and also to make the difference between the two as small as possible. Equation 29 represents the calculation process for evaluating criterion F1.


(29) $$F1=2\times \frac{P\times Recall}{P+Recall}$$

where *P* is precision.

### 5.4 Experimental results

#### 5.4.1 Object detection benchmark experiment based on Faster R-CNN

The primary objective of this experiment is to assess the impact of different feature extraction networks on object detection. In the end, 16 backbones are selected to evaluate the overall performance of the Faster R-CNN. The backbones fall into 10 main types: ResNet (He et al., 2016), Res2Net (Gao et al., 2019), ResNeSt (Zhang et al., 2022), RegNet (Radosavovic et al., 2020), HrNet (Sun et al., 2019), HarDNet (Chao et al., 2019), EfficientNet (Tan and Le, 2019), MobileNetV2 (Sandler et al., 2018), VoVNet (Lee et al., 2019), and Swin Transformer (Liu et al., 2021b). While the experiments are conducted using two different frameworks, Detectron2 and MMDetection, the parameters remain consistent. **Table 5** shows the parameter settings used in the current experiment, and undisclosed parameters use the default settings of the respective frameworks.

In the first stage of object detection, the size of the input image is not fixed, but we normalize the size of the image in the dataset. This means that the processed image has a moderate aspect ratio and sharp pixels. Before extracting the image features in the initial stage, we standardize the image size to 448 × 448 and extract 1 × 1,024 feature tensors using the feature extractor. These are then saved in an npy file for use in subsequent image caption generation tasks. It should be noted that there will be multiple diseased regions in an image, and each diseased region will generate a 1 × 1,024 tensor. Therefore, there will be several tensors in the.npy file corresponding to an image. The Faster R-CNN model used to extract image features utilizes the Swin Transformer as its backbone. In the Swin Transformer, the default stride of the convolution is set to 2, and the size of the convolution kernel is 3 × 3. This is because in the Swin Transformer Block, the convolutional kernel size of each 2D convolutional layer is set to 3 × 3 for local feature extraction. It is important to note that the size of the convolutional kernel can be adjusted based on the specific task and dataset to achieve optimal performance. In the Swin Transformer, using a 3 × 3 size as the default is a common choice, but it can be adjusted as necessary. Specifically, in a Swin Transformer Block, a 2D convolutional layer typically employs a convolution operation with a stride of 2. Using a two-step convolution can effectively decrease the size of the feature map and reduce the computational workload. This configuration is used in the Swin Transformer to achieve a balance between chunk processing and attention mechanisms, leading to improved performance and results. The activation function used in the encoder–decoder is ReLU, which enhances the expressive capability of the features through non-linear transformation. It strengthens the part with larger values and suppresses the part with smaller values, resulting in better expression of the relevant features.

**Tables 7**, **8** show the experimental results under the Detectron2 and MMDetection frameworks, respectively. Finally, the Swin Transformer is selected for feature extraction. Swin Transformer adopts a hierarchical structure, creating layered representations by initially using small-sized patches and gradually merging adjacent patches into deeper layers of the Transformer. When *IoU* = 0.5:0.05:0.95, *mAP* is 0.674. When *IoU* = 0.5, *mAP*_50_ is 0.862. When *IoU* = 0.75, *mAP*_75_ is 0.793. The results indicate that, among the tested backbones, the Swin Transformer performs the best. Moreover, the experimental results show that there is no absolute linear upward relationship between the backbone performance and parameter scale, and the appropriate parameter scale should be analyzed according to the specific use scenario. For example, ResNet-101 and ResNet-50 have *mAP*_50_ values of 0.771 and 0.788, respectively, indicating a proportional relationship between backbone performance and parameter scale. However, HarDNet-68 and HarDNet-39 achieve *mAP* values of 0.679 and 0.729, respectively, suggesting an inversely proportional relationship between backbone performance and parameter scale.

**Table 7:** Faster R-CNN benchmark experiment (Detectron2 framework).

Framework	Backbone	*mAP*	*mAP*_50_	*mAP*_75_
Faster R-CNN	HarDNet-68	0.472	0.679	0.514
	HarDNet-39	0.469	0.729	0.506
	EfficientNet-B4	0.544	0.781	0.640
	EfficientNet-B0	0.532	0.740	0.618
	MobileNetV2	0.511	0.734	0.600
	VoVNet-19	0.625	0.792	0.733
	VoVNet-99	0653	0.812	0.763
	Swin Transformer	0.674	0.862	0.793

**Table 8:** Faster R-CNN benchmark experiment (MMDetection framework).

Framework	Backbone	*mAP*	*mAP*_50_	*mAP*_75_
Faster R-CNN	ResNet-101	0.580	0.771	0.671
	ResNet-50	0.585	0.788	0.694
	Res2Net-101	0.582	0.772	0.682
	RegNetX-3.2GF	0.580	0.798	0.683
	ResNeSt-50	0.525	0.746	0.597
	ResNeSt-101	0.609	0.821	0.705
	HrNet w-18	0.584	0.782	0.649
	HrNet w-32	0.599	0.782	0.691

#### 5.4.2 Quantitative and qualitative analyses of DIC-Transformer

**Figure 7:**
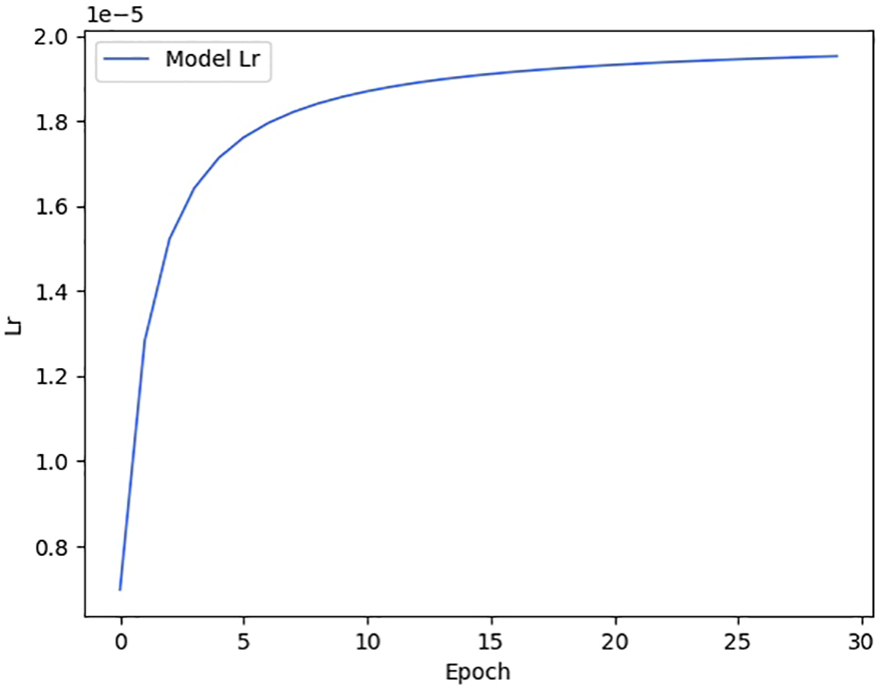
Model learning rate change curve.

**Figure 8:**
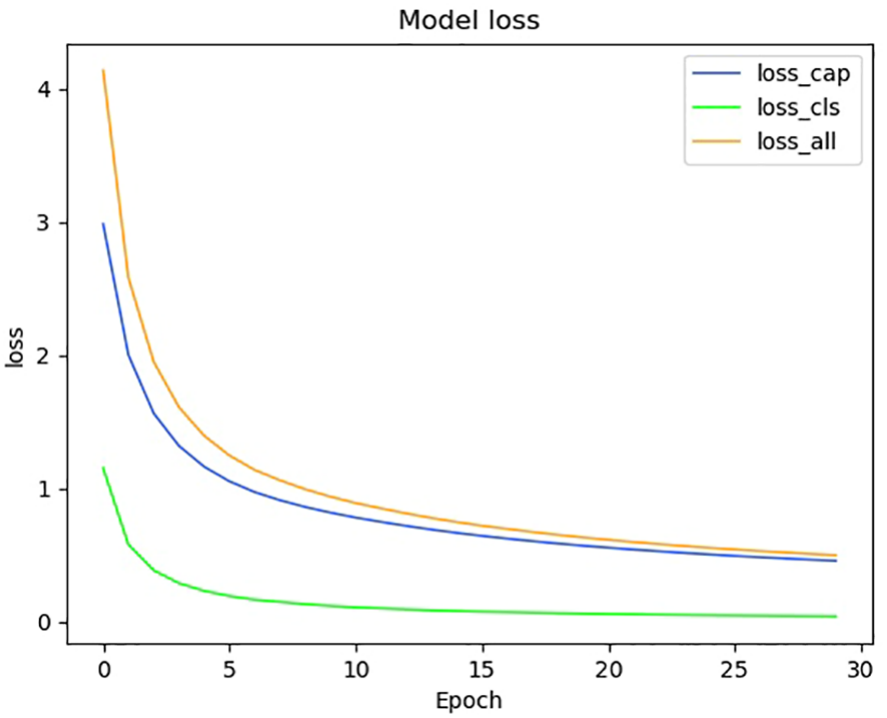
Model loss change curve.

We show the changes in various information during the model training process in the form of a line chart. **Figure 7** shows the change process of learning rate during model training. **Figure 8** shows the changes in the three losses in the training process, which are the loss changes in the image caption generation module, the loss changes in the image classification module, and the total loss changes in the model. **Figure 3** shows the variation curves of various evaluation indicators of image classification and image subtitle generation results.

##### 5.4.2.1 DIC-Transformer image caption generation performance test

This is followed by a simple description of the eight image caption generation models to be compared. AoANet (Huang et al., 2019) introduces a new multi-level attention mechanism to enhance image caption generation by incorporating attention weighting in an attention weight. UpDown (Anderson et al., 2018) introduces underlying and top-level attention mechanisms, proposes trainable underlying features, and combines the attention mechanism with the language model. Adaatt (Lu et al., 2017) introduces visual flags to guide the allocation of attention, proposes an adaptive attention mechanism, and builds an end-to-end generative model. ShowTell (Vinyals et al., 2015) utilizes attention mechanisms and employs Multilayer Perceptron (MLP) as generators, combining CNN and LSTM. FC (Rennie et al., 2017) uses a CNN-encoded image as input to predict the second word. Instead of using a static spatial pool representation of the image, the attention model in Att2in (Dosovitskiy et al., 2020) dynamically reweights the input space (CNN) feature to focus on a specific area of the image at each time step. *M* ^2^ Transformer (Cornia et al., 2020) integrates prior knowledge of learning, learns multi-level representations of relationships between image regions, and uses mesh connections to leverage low-level and high-level features during the decoding stage. DLCT (Luo et al., 2021) enhances contextual information and fine-grained details through a new bidirectional self-attention (DWSA) and a locally constrained cross-attention module. ExpansionNet v2 (Hu et al., 2022) explores the potential performance bottlenecks in input length in deep learning methods.

LATGeO (Dubey et al., 2023a) proposes a novel attention mechanism that is capable of efficiently processing geometrically related objects in images when generating image descriptions. This method not only considers global information but also pays attention to the spatial relationship between different objects in the image through a fine-grained local attention mechanism. The proposed framework, label-attention transformer with geometrically coherent objects (LATGeO (Dubey et al., 2023b)) acquires proposals of geometrically coherent objects using a deep neural network (DNN) and generates captions by investigating their relationships using label attention module (LAM). Stack-Captioning (Gu et al., 2018), a coarse-to-fine multi-stage prediction framework for image captioning, is composed of multiple decoders, each of which operates on the output of the previous stage, producing increasingly refined image descriptions. A new diffusion model-based paradigm tailored for image captioning is proposed, namely, Semantic-Conditional Diffusion Networks (SCD-Net; Luo et al., 2023), which breaks the deeply rooted conventions in learning Transformer-based encoder–decoder.

Next, we test the performance of the DIC-Transformer and the above model on the image classification task based on ADCG-18. The criteria used to evaluate the model are Bleu1, Bleu2, Bleu3, Bleu4, CiderD, and Rough, which are common in the field of caption generation. Finally, **Table 9** shows the test results of seven classic caption generation models and the DIC-Transformer model.

**Table 9:** Comparison results of DIC-Transformer and classic image caption generation models.

Method	Bleu1	Bleu2	Bleu3	Bleu4	CiderD	Rough
Fc	0.746	0.535	0.364	0.249	420.96	0.707
Att2in	0.712	0.466	0.242	0.159	342.95	0.637
DLCT	0.721	0.502	0.348	0.293	434.54	0.664
LATGeO	0.682	0.472	0.305	0.214	335.26	0.636
Stack-Captioning	0.706	0.495	0.335	0.257	362.24	0.669
UpDown	0.679	0.489	0.316	0.228	383.40	0.657
SCD-Net	0.714	0.451	0.253	0.161	351.24	0.640
AoANet	0.727	0.539	0.353	0.280	431.09	0.677
*M* ^2^ Transformer	0.738	0.543	0.378	0.271	433.71	0.685
ExpansionNet v2	0.741	0.549	0.386	0.287	437.09	0.708
LATGeO	0.739	0.531	0.363	0.278	432.12	0.701
Ours	0.756	0.561	0.404	0.294	450.51	0.721

In this paper, there are seven comparison methods used for image caption generation tasks, which are roughly divided into two categories: one is the top-down method, and the other is a bottom-up approach. Experiments show no significant difference between top-down and bottom-up approaches. For example, the ShowTell method exhibits the worst results in the top-down method, whereas the Fc method performs the best effect, showing little difference from the optimal method in the bottom-up method. The model with the highest CiderD score is AoANet, with a value of 431.09. However, the model presented in this paper achieves a CiderD score of 450.51, which is 19.42 points higher than the CiderD score of AoANet.

##### 5.4.2.2 DIC-Transformer classification performance test

The purpose of this experiment is to demonstrate that DIC-Transformer is superior to other classification models in terms of classification performance. To compare with classical classification methods, DIC-Transformer is evaluated on ADCG-18, and the results are shown in **Table 10**. Under the context of error propagation (where the region detection module is trained solely on the training set), the classical CNN model with the best classification performance is MobilenetV2, achieving an Acc value of 0.830. In contrast, DIC-Transformer achieves an Acc value of 0.854, which is 0.024 higher than the Acc of MobilenetV2. In addition, in the absence of error propagation (where the region detection module is trained using both the training and testing sets), the DIC-Transformer’s ACC value increases by an additional 0.031, resulting in an Acc value of 0.885. The results show that in the classification performance comparison experiment using the ADCG-18, DIC-Transformer outperforms other classical classification models, showing a better classification effect.

**Table 10:** Comparison results of DIC-Transformer and classical CNN models.

Method	Acc	Recall	F1
AlexNet, Krizhevsky et al. (2012)	0.743	0.641	0.652
ResNet-101	0.827	0.779	0.786
EfficientNet	0.814	0.775	0.778
VggNet, Simonyan and Zisserman (2014)	0.772	0.698	0.705
GoogleNet, Szegedy et al. (2015)	0.754	0.683	0.695
InceptionV3, Szegedy et al. (2016)	0.717	0.640	0.653
MobileNetV2	0.830	0.776	0.778
RepVGG, Ding et al. (2021)	0.793	0.705	0.712
GhostNet, Han et al. (2020)	0.752	0.685	0.689
SqueezeNet, Koonce and Koonce (2021)	0.784	0.725	0.732
Ours w/EP	0.854	0.854	0.853
Ours w/o EP	0.885	0.885	0.886

##### 5.4.2.3 Ablation experiment of DIC-Transformer

In this set of ablation experiments, the main purpose is to verify the contribution of each module in the model. The modules involved in the experiment include the patch embedding module, the region detection module, the caption generation module, and the position encoding module. The results are shown in **Table 11**. In Experiments I and II, the region detection module is not used, and the image is divided into 16 * 16 and 32 * 32 grid input to the sequence encoding module. This method of dividing images comes from VIT (Dosovitskiy et al., 2020), called the PetchEmbedding method. In Experiment III, the visual feature vector outputted by the region detection module is used as the input to the sequence encoding module. The output of the sequence encoding module is then fed into the caption generation module to obtain the visual vector weighted by text features. Finally, this vector is used as the input of the classification module to obtain the category label of the disease. In addition, the positional coding module is used in the sequence encoding module. Experiment IV is based on Experiment III but without a position coding module. Experiment V removes the caption generation module from the setup in Experiment IV, and the visual feature vectors generated by the sequence encoding module are directly inputted to the classification module to classify the image. Experiments VI and VII remove the caption generation module from the setup in Experiments I and II, respectively. They directly input the visual feature vectors obtained by the segmented image processed by the sequence encoding module into the classification module to obtain the category label of the image.

**Table 11:** In the presence of error propagation, the effect of a single module on model performance results.

Ablation					Image caption						Classification			Index
P16	P32	Detection	Describe	Position	Bleu1	Bleu2	Bleu3	Bleu4	CiderD	Rouge	Acc	Recall	F1	
✓	✓		✓		0.612	0.443	0.285	0.204	404.67	0.537	0.714	0.714	0.711	I
			✓		0.632	0.487	0.313	0.224	413.25	0.583	0.786	0.786	0.782	II
		✓	✓		0.722	0.539	0.389	0.283	430.71	0.678	0.837	0.837	0.834	III
		✓	✓	✓	0.756	0.561	0.404	0.295	450.51	0.721	0.854	0.854	0.853	IV
		✓		✓	–	–	–	–	–	–	0.846	0.846	0.844	V
✓					–	–	–	–	–	–	0.478	0.478	0.467	VI
	✓				–	–	–	–	–	–	0.425	0.425	0.416	VII

The ✓ in the table means that the model in the experiment contains the current module, and the model in the experiment does not contain the current module without the ✓ symbol.

Experiments I, II, and III are conducted to verify the influence of the region detection module on the overall model performance, as shown in **Table 11**. Taking Bleu as an example, the average values of Experiments I and II are 0.612 and 0.632, respectively, while the average value of Experiment III is 0.722. The latter is 0.11 and 0.09 higher than the average values of the previous two experiments. This difference is due to the image meshing method used in Experiments I and II, which results in the divided images containing much background information. Additionally, Experiments I and II demonstrate that meshing the image into 32 * 32 works better than dividing it into 16 * 16 grids. Notably, location information is utilized in both experiments. Experiments III and IV evaluate the effect of the position coding module in the sequence encoding module on the overall model performance. In Experiment IV, the bounding box coordinates of the disease area in the image are used as location encoding. The mean value of Bleu1 in Experiment IV using position coding is 0.756, whereas the mean value of Bleu1 in Experiment III without position coding is 0.722, resulting in a difference of 0.034. This indicates that using bounding box coordinates as position coding has a positive impact on the model’s performance. Experiments IV and V evaluate the influence of the caption generation module on classification performance. Taking Acc as an example, the classification result of the model containing the caption generation module is 0.854, while the classification result of the model without the caption generation module is 0.846. The results indicate that the introduction of the caption generation module to weigh visual feature vectors with text features can improve classification performance. In addition, in both experiments, location information is used. Experiments VI and VII remove the caption generation module based on Experiments I and II, respectively. The results show that the classification performance is significantly reduced, with the classification accuracy being 0.478 and 0.425, respectively.

Finally, the influence of error propagation on model performance in DIC-Transformer is quantitatively analyzed. In effect, all the images in the dataset are used as the training set for the first stage disease region detection module. This eliminates errors caused by the region detection module. Next, the trained disease area detector is used to obtain the feature information and disease area location details of the image. The ablation experiment is then repeated using the obtained data. The results of this experiment are summarized in **Table 12**. The mean accuracy in the final results of Experiments X, XI, and XII is 0.875, while Experiments VIII, IX, XIII, and XIV are consistent with the results presented in **Table 11**, as they do not involve the region detection module.

**Table 12:** In the absence of error propagation, the effect of a single module on model performance results.

Ablation					Image caption						Classification			Index
P16	P32	Detection	Describe	Position	Bleu1	Bleu2	Bleu3	Bleu4	CiderD	Rouge	Acc	Recall	F1	
✓	✓		✓		0.612	0.443	0.285	0.204	404.67	0.537	0.714	0.714	0.711	VIII
			✓		0.632	0.487	0.313	0.224	413.25	0.583	0.786	0.786	0.782	IX
		✓	✓		0.764	0.583	0.411	0.325	478.56	0.729	0.862	0.862	0.8	X
		✓	✓	✓	0.7778	0.599	0.416	0.346	490.22	0.74	0.885	0.885	0.886	XI
		✓		✓	–	–	–	–	–	–	0.877	0.877	0.877	XII
✓					–	–	–	–	–	–	0.478	0.478	0.467	XIII
	✓				–	–	–	–	–	–	0.425	0.425	0.416	XIV

The ✓ in the table means that the model in the experiment contains the current module, and the model in the experiment does not contain the current module without the ✓ symbol.

##### 5.4.2.4 Case study of the DIC-Transformer

**Figure 9:**
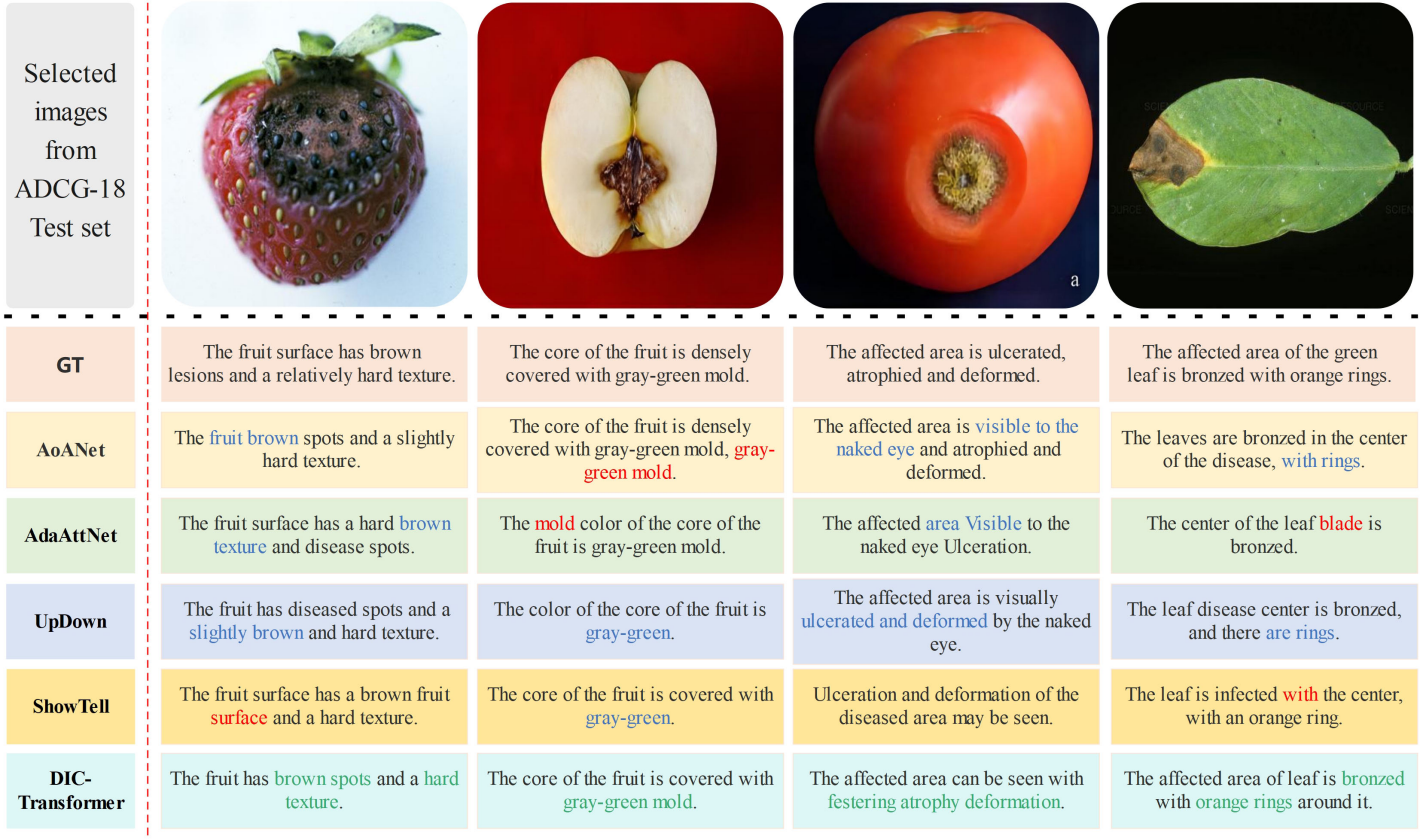
Comparison of caption generation results with real caption.

To demonstrate the superiority of the proposed DIC-Transformer in the field of image caption generation, we compare it with the AoANet, AdaAtt, UpDown, and ShowTell models. The caption generation results of each model are shown in **Figure 9**. It should be noted that the image caption dataset used in the experiment is Chinese, and the statements in the case study are translated into English for ease of understanding. The red part of the figure represents the wrong word, the blue part represents the missing word or the wrong combination of words and other inaccurate results, and the green part represents the correct disease keyword. Based on the four examples in **Figure 9**, we perform a meticulous analysis of the captions generated by each model, and the results are as follows:

1. As illustrated in **Figure 9**, the captions generated by the other four models lack relevant disease feature keywords. Specifically, the models AoANet and UpDown do not generate the color of the rings, the model AdaAttNet does not include any ring-related statements, and the model ShowTell omits the color of the disease area.
2. In the case of apple mold heart disease shown in **Figure 9**, the image caption generated by the model AoANet repeats the phrase “gray-green mold” twice, and the model AdaAttNet also repeats the word “mold” twice, both of which result in semantic confusion. Although both UpDown and ShowTell did not have duplicate issues, they both lacked the keyword “mold”.
3. In the last image, the caption generated by models AOANet and UpDown lacks color information. The model AdaAttNet generates words with repetitive meanings in captions. The model ShowTell generates the wrong word “with”.

However, the DIC-Transformer model we build generates comprehensive keywords and fluent sentences. The above experimental results can fully prove the superiority of the DIC-Transformer in image caption generation. In order to more fully evaluate the quality of the generated statements, we invite experts or volunteers to perform a manual evaluation of the generated statements. This can be carried out through subjective scoring or preference sorting. By gathering opinions and feedback from evaluators, we can determine the advantages of the utterances generated by our model over other methods in terms of readability, fluency, and accuracy. In addition to expert assessments, we also conduct user surveys to gain a broader perspective. By inviting real users to rate and rank the generated statements, we can obtain more feedback that verifies the superiority of the statements generated by our model in terms of user experience. Through the above statistical analysis methods, we can objectively prove the superiority of the statements generated by our model over other methods.

##### 5.4.2.5 Summary of the comparison results between DIC-Transformer and other methods

In this paper, we conduct a total of three experiments, and the specific results of the experiments are as follows.

The first experiment is the Faster R-CNN benchmark experiment, which aims to select an optimal backbone for Faster R-CNN. Specifically, we select 16 different backbones based on the dataset to test the performance of Faster R-CNN in detecting disease regions, and the experimental results show Swin Transformer works best as backbones.

In the second experiment, the DIC-Transformer is compared with seven existing image caption generation models. The experimental results in **Table 7** show that the model proposed by us based on ADCG-18 performs well in the field of image caption generation, and the performance is due to the other seven existing models. First, we consider the caption generation accuracy of the model. Compared to other models, DIC-Transformer can more accurately generate caption descriptions that match the content of the image. Second, we evaluate the language fluency and sentence quality of the model. The results show that the captions generated by DIC-Transformer are more natural and smooth, and the sentence structure is more reasonable and coherent. In contrast, other models may have some imperfections or non-conform to grammatical rules in terms of language expression.

**Figure 10:**
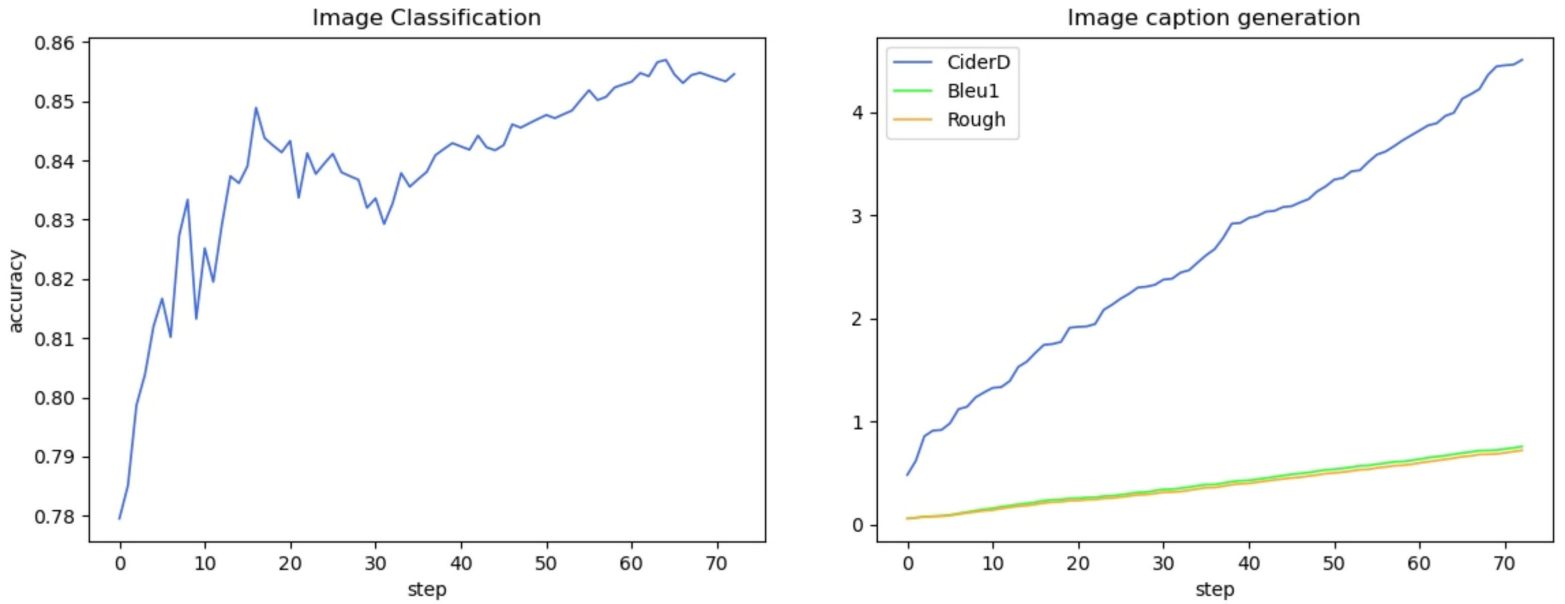
The change curve of evaluation indicators related to image classification and image caption generation.

The third experiment aims to test the classification performance of the DIC-Transformer based on ADCG-18. We compare the DIC-Transformer with seven classical CNN models, and the results show that the classification performance of the DIC-Transformer is better than that of other existing models. **Figure 10** shows the variation curves of the evaluation indicators related to image classification and image caption.

In summary, our experimental results show that our proposed method has better performance than other methods in different tasks. This provides broad prospects for the application of our method in areas such as caption generation and image classification.

To evaluate the results of image caption generation, it is necessary to comprehensively consider the indicators of accuracy, completeness, semantic consistency, naturalness, and information richness and combine the experimental results for objective evaluation. The quantitative experimental results prove the accuracy of the model, and the indicators such as completeness and information richness of the generated sentences are also superior in the qualitative analysis section.

## 6 Conclusion

In this paper, we propose a model called DIC-Transformer, which is capable of generating explanatory textual descriptions corresponding to the classification results of agricultural plant diseases. Then, we collect a dataset called ADCG-18, which includes images of 18 diseases and their corresponding textual descriptions. We conduct numerous experiments on the proposed method from multiple angles on this constructed dataset to demonstrate the method’s superiority. For the two challenges presented in the Introduction section, the experimental results indicate that the identification accuracy of DIC transformers reaches 85.4% in a relatively small sample, which is 2.4% higher than that of the model with the best performance based on CNN. This demonstrates that the DIC-Transformer effectively addresses the first challenge of enhancing image classification performance in the context of limited data volume. In response to the second challenge, we introduced technology for generating image captions. The technology can generate descriptive information based on images, similar to how agricultural experts describe plant diseases. The results of ablation experiments show that combining image caption generation and image classification technology can improve the accuracy of image classification. Based on ADCG-18, the results of the six evaluation indexes Bleu1, Bleu2, Bleu3, Bleu4, Cider-D, and Rouge of the proposed DIC-Transformer model are 0.756, 0.561, 0.404, 0.294, 450.51, and 0.721, respectively. These results indicate that the model outperforms other models.

Image caption generation technology can accurately describe the diseases affecting crops, enhance the precision of disease identification, aid in early detection and diagnosis of plant diseases, and minimize crop losses. It can also be integrated with other agricultural intelligence technologies, such as drones and sensors, to achieve automatic monitoring and management of farmland. This integration can enable farmers and agricultural practitioners to accurately identify diseases through image capture, even without expertise in disease identification. This will help reduce the barrier to identifying diseases and promote the adoption and application of agricultural technology.

However, the model we propose still has certain limitations. The DIC-Transformer is a two-stage model that suffers from the issue of error propagation, as depicted in **Figure 10**. For instance, a distinctive characteristic of peanut leaves is their green color. If the first-stage region detection module fails to recognize this aspect, the second-stage caption generation module will not generate a corresponding caption. In our upcoming work, we intend to integrate the initial stage of the feature extraction module with the subsequent caption generation and classification modules to create a comprehensive model. This will help prevent some of the issues that arise with two-stage models and improve their performance.

## Data availability statement

The raw data supporting the conclusions of this article will be made available by the authors, without undue reservation.

## Author contributions

QZ: Funding acquisition, Writing – original draft, Writing – review & editing. JS: Methodology, Validation, Writing – original draft. SW: Investigation, Software, Writing – review & editing.
